# Hierarchical classification of acute and chronic osteoporotic vertebral compression fractures of the lumbar spine using X-ray images

**DOI:** 10.1007/s00256-026-05328-7

**Published:** 2026-08-11

**Authors:** Joohyun Kim, Keewon Shin, Sungjae An, Subum Lee, Woo-Keun Kwon, Younggyu Oh, Kyu-Chong Lee, Sungjin Park, Kyung-Sik Ahn, Junseok W. Hur

**Affiliations:** 1 Department of Neurosurgery, Korea University Anam Hospital, Korea University College of Medicine, Seoul, Republic of Korea; 2 Department of Convergence Medicine, AMIST, Asan Medical Center, University of Ulsan College of Medicine, Seoul, Republic of Korea; 3 Department of Neurosurgery, Korea University Guro Hospital, Korea University College of Medicine, Seoul, Republic of Korea; 4 Department of Radiology, Korea University Anam Hospital, Korea University College of Medicine, Seoul, Republic of Korea; 5 Institute of Human Behavior and Genetics, Korea University College of Medicine, Seoul, Republic of Korea; 6 Advanced Medical Imaging Institute, Korea University College of Medicine, Seoul, Republic of Korea; 7 National Research Laboratory for Convergence Degradation Biology, Korea University, Seoul, Republic of Korea; 8 Center for Genomic Research and Development, Korea University Anam Hospital, Seoul, Republic of Korea; 9 GeneCker Co., Ltd., Seoul, Republic of Korea

**Keywords:** Deep learning, Hierarchical classification, Osteoporotic vertebral compression fractures, Lumbar spine, X-ray imaging

## Abstract

**Objective:**

To develop and validate a hierarchical deep learning model for differentiating acute and chronic lumbar osteoporotic vertebral compression fractures (OVCFs) using X-ray images.

**Materials and methods:**

We retrospectively reviewed approximately 2600 lateral lumbar radiographs obtained from patients clinically suspected of having OVCFs between 2007 and 2022. After excluding poor-quality images and surgically instrumented vertebrae, 1299 radiographs (6495 vertebral patches, L1–L5) were included. Labeling was performed by neurosurgeons and radiologists using X-ray images, with CT and/or MRI findings serving as the reference standard. A two-step hierarchical classification was implemented: first classifying vertebrae into Normal-Chronic, Acute, and Indeterminate (cement-augmented vertebrae without instrumentation) groups, followed by subdivision of the Normal-Chronic group into Normal and Chronic categories.

**Results:**

A total of 1299 radiographs were evaluated. The hierarchical model achieved an accuracy of 91% in the initial three-class step. For the detection of acute fractures in the final classification step, the model demonstrated a sensitivity of 91.0% (95% CI 84.8–95.0%), a specificity of 82.1% (95% CI 79.8–84.5%), and a high negative predictive value (NPV) of 98.8% (95% CI 97.9–99.3%). The Normal-aligned hierarchical approach outperformed the Acute-aligned and end-to-end models, particularly for acute and chronic cases.

**Conclusion:**

The proposed hierarchical approach enhances the diagnostic utility of standard X-ray images by enabling more accurate classification of lumbar fracture types. This study is limited by its single-institution retrospective design. This model may reduce reliance on advanced imaging and support faster and more informed clinical decision-making.

## Introduction

The distinction between acute and chronic osteoporotic vertebral compression fractures (OVCFs) is of high clinical significance, as it directly influences treatment strategies and patient outcomes. Acute OVCFs often require urgent intervention, orthosis application, and aggressive osteoporosis treatment to stabilize the spine, whereas chronic OVCFs are generally managed with pain control and physical therapy. Misclassification can lead to inappropriate management, exacerbating patient morbidity and increasing healthcare costs [1].

Diagnosis of OVCFs typically relies on X-rays, computed tomography (CT), and magnetic resonance imaging (MRI). X-rays are widely used due to their accessibility but primarily detect the presence of fractures without distinguishing acuity. CT provides detailed bony architecture and identifies fracture acuity through findings such as cortical disruption. MRI remains the gold standard, detecting bone marrow edema in acute cases and fatty marrow replacement in chronic cases through signal intensity changes [2].

Several studies have demonstrated the utility of CT and MRI in distinguishing acute from chronic OVCFs. CT is particularly adept at identifying acute OVCFs through features such as recent cortical breaks or callus formation indicative of healing in chronic OVCFs [2]. For instance, Zhu et al. implemented a deep learning framework using convolutional neural networks (CNNs) to automatically analyze CT features, improving diagnostic precision and minimizing observer variability. Dual-energy CT (DECT) further enhances bone marrow edema detection by leveraging virtual noncalcium techniques to highlight edema-associated density changes, offering diagnostic performance comparable to MRI when read by experienced radiologists [3]. MRI utilizes advanced sequences such as T1-weighted imaging and short tau inversion recovery (STIR) to identify signal intensity changes, distinguishing acute edema from chronic fatty marrow replacements [4]. Hu et al. integrated a machine learning model with MRI data to predict subsequent fracture risks, emphasizing the value of advanced feature extraction in clinical applications.

In contrast, while X-ray imaging is ubiquitous in clinical settings, it lacks the specificity to distinguish acute from chronic OVCFs. Although effective in detecting fracture presence and deformity, its limited resolution and inability to capture soft tissue or marrow changes render it unsuitable for acuity assessment. Some studies have explored deep learning methods to detect general OVCFs on X-rays [5–9], and recent approaches have attempted to distinguish acute from chronic fractures using radiomics features fused with clinical data [10]. However, to date, no study has presented a purely X-ray-based classification model with a hierarchical design that reflects real-world diagnostic decision-making.

To address these limitations, we propose a hierarchical deep learning classification model based on conventional X-ray imaging. Our normal-aligned strategy utilizes CNNs to enhance the interpretive capabilities of radiographs. By implementing a two-step classification process, the model aims to supplement initial radiographic assessment when advanced imaging is unavailable. Unlike prior studies integrating CT, MRI, or clinical variables, our approach operates solely on lateral radiographs, allowing direct integration into radiology workflows. While its performance does not surpass MRI or CT, it is among the highest reported for X-ray–only methods, suggesting its utility in resource-limited clinical settings.

## Materials and methods

### Datasets

This study was approved by the Institutional Review Board of Korea University Anam Hospital (IRB No. 2023AN0268) and was conducted in accordance with the principles of the Declaration of Helsinki. The requirement for written informed consent was waived due to the retrospective nature of the study.

We retrospectively reviewed approximately 2600 lateral lumbar radiographs obtained from patients who were clinically suspected of having OVCFs between 2007 and 2022 at our institution. Each image included five lumbar vertebrae (L1–L5), and all patient data were fully anonymized before analysis.

After quality assessment and eligibility screening, 1299 radiographs were finally included for model development. The inclusion criteria were as follows: (1) availability of standard lateral lumbar radiographs with clear visualization of vertebral bodies; (2) confirmed diagnosis of acute or chronic OVCF based on CT or MRI reports; and (3) absence of spinal instrumentation involving metallic implants. Exclusion criteria included the following: (1) incomplete or poor-quality images (motion artifact, occlusion, or unclear vertebral borders); (2) severe traumatic or pathologic fractures unrelated to osteoporosis. Cases with lumbosacral transitional anatomy were managed by assigning vertebral levels based on counting from the T12–L1 junction.

This study specifically focused on lumbar vertebrae (L1–L5) due to their clinical and diagnostic significance. The lumbar spine is particularly susceptible to OVCFs compared to the thoracic spine, primarily due to its biomechanical role in weight-bearing and mobility, which exposes it to greater mechanical stress [11, 12]. Additionally, the larger and more uniform morphology of lumbar vertebrae, in contrast to the smaller and more variable structures of the thoracic spine, simplifies segmentation and enhances model accuracy [13]. Moreover, the absence of overlapping rib shadows in lumbar radiographs minimizes visual interference, enabling more precise boundary delineation and facilitating accurate identification of fracture morphology.

Following exclusion, 1299 radiographs were included, comprising 722 cases clinically diagnosed with acute fractures and 577 cases classified as chronic or normal. The baseline characteristics of the study population are summarized (Table 1). This dataset yielded 6495 annotated vertebral patches for analysis. All labeling was performed manually by seven experienced clinicians (five neurosurgeons and two radiologists) using Aview software (Coreline Soft) to delineate the anterior and endplate boundaries of each vertebral body using polygon-based point annotation. Fracture classification was determined based on morphological assessment of X-ray images, with CT and/or MRI reports serving as the definitive reference for each case. Potential inter-rater variability was minimized as fracture classification was referenced against CT and/or MRI reports for all cases.

**Table 1:** Baseline characteristics of the patients corresponding to the included radiographs (n = 1299 radiographs)

Variables	Values
Age, years	67.7 ± 14.7
≤ 59	303 (23.3%)
60–69	289 (22.2%)
70–79	459 (35.3%)
≥ 80	248 (19.1%)
Sex	
Men	421 (32.4%)
Women	878 (67.6%)
Comorbidities	
Hypertension	617 (47.5%)
Diabetes	257 (19.8%)
BMD^†^ (T-scores)	
Spine	−2.43 ± 1.29
Hip	−2.31 ± 1.14
Lowest	−2.80 ± 1.06
Clinical features	
Previous fracture history	35 (2.7%)
Prior osteoporosis therapy	25 (1.9%)
The time to intervention (days)	26.9 ± 73.8
Treatment	
Vertebroplasty	84 (6.5%)
Kyphoplasty	274 (21.1%)

Plus-minus values represent means ± standard deviation

^†^Lowest BMD values were based on dual-energy X-ray absorptiometry (DXA) of the proximal femur and lumbar spine, available for 967 patients. DXA was not required as an inclusion criterion, and BMD data were not incorporated into model development or analysis

**Fig. 1:**
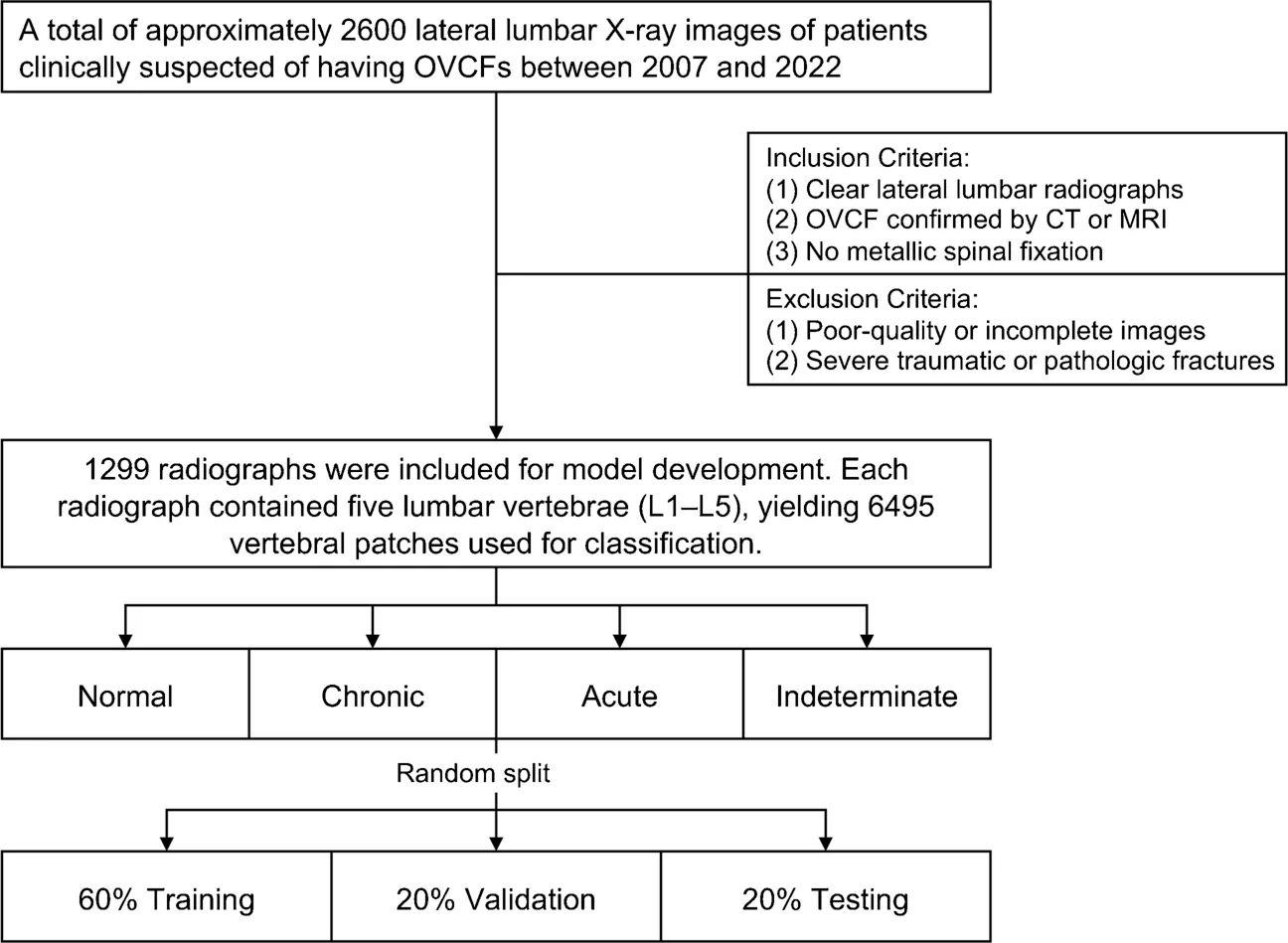
Flowchart of dataset selection and preprocessing. Approximately 2600 lateral lumbar X-ray images from patients suspected of having OVCFs were collected between 2007 and 2022. After quality control and eligibility screening, 1299 radiographs were included for model development. Each radiograph provided five lumbar vertebrae (L1–L5), resulting in a total of 6495 vertebral patches. These were manually labeled as normal, chronic, acute, or indeterminate (cement augmentation without metallic fixation) for classification. The dataset was randomly divided into training (60%), validation (20%), and testing (20%) sets

In addition to the normal, chronic, and acute categories, an indeterminate group was defined for vertebrae that had undergone cement augmentation (vertebroplasty or kyphoplasty) without metallic fixation, representing post-treatment cases not clearly distinguishable by morphology alone. To ensure a consistent model training process, the dataset was randomly partitioned on a per-patient basis into three subsets: training (60%), validation (20%), and testing (20%). The dataset selection process is summarized (Fig. 1), and the distribution of labeled vertebrae by spinal level and fracture category is provided (Table 2).

**Table 2:** Summary of the lumbar vertebral dataset by spinal level and fracture category

Level	Normal	Chronic	Acute	Indeterminate	Total
L1	689 (53.0%)	64 (4.9%)	282 (21.7%)	264 (20.3%)	1299
L2	862 (66.4%)	108 (8.3%)	166 (12.8%)	163 (12.5%)	1299
L3	1146 (88.2%)	54 (4.2%)	45 (3.5%)	54 (4.2%)	1299
L4	1126 (86.7%)	60 (4.6%)	53 (4.1%)	60 (4.6%)	1299
L5	1167 (89.8%)	68 (5.2%)	30 (2.3%)	34 (2.6%)	1299
Total	4990 (76.8%)	354 (5.5%)	576 (8.9%)	575 (8.9%)	6495

Each of the 1299 radiographs provided five lumbar vertebrae (L1–L5), providing a total of 6495 vertebral patches for hierarchical classification

### AI models

**Fig. 2:**
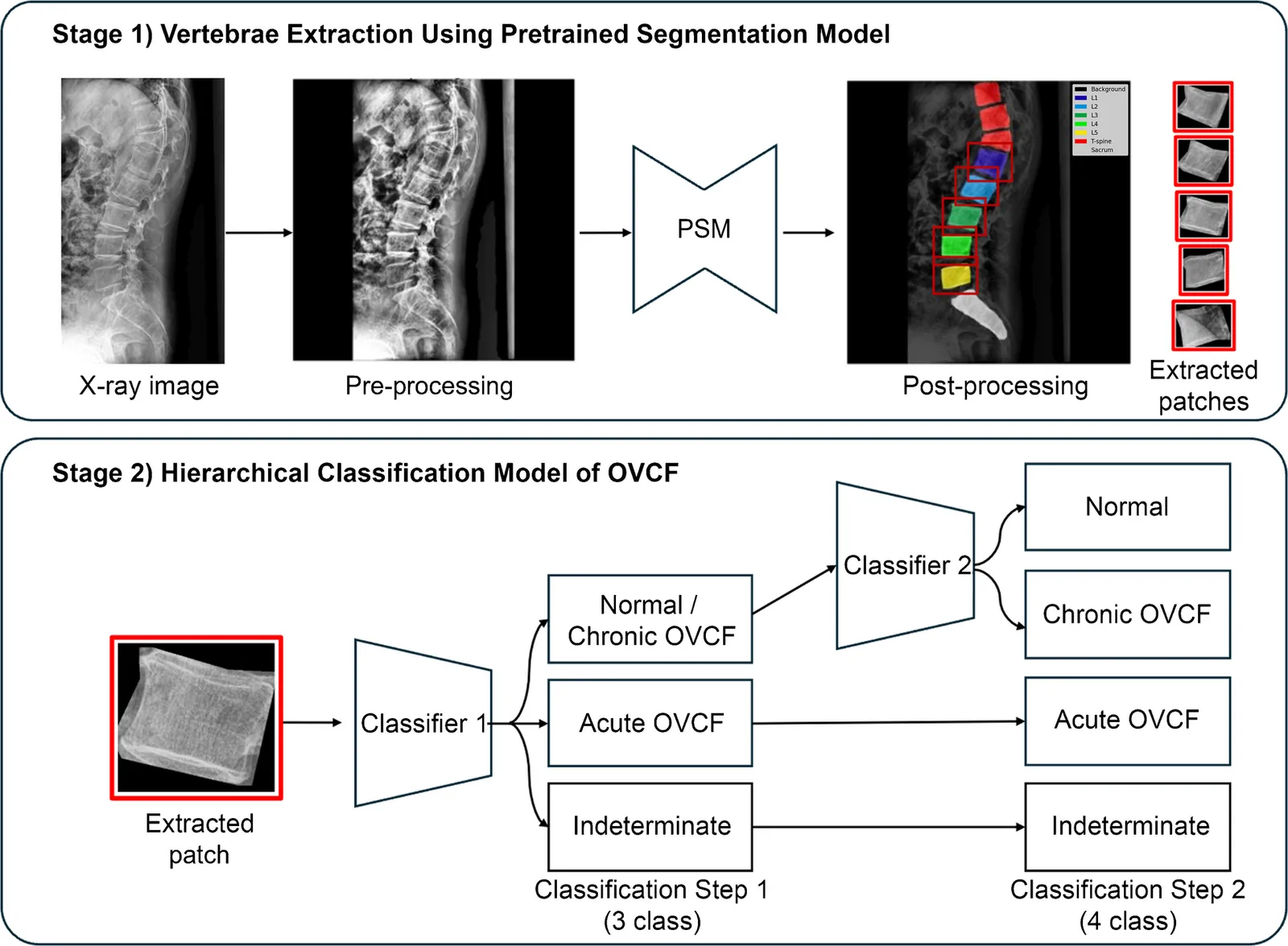
Overview of the proposed framework. Schematic of the study workflow, comprising vertebrae extraction from lateral X-ray images followed by a two-step hierarchical classification process. A pretrained segmentation model originally developed for thoracic-to-sacral vertebrae was used for extraction; however, only lumbar vertebrae (L1–L5) were utilized for classification

The proposed methodology consists of two main stages: (1) vertebrae extraction from radiographic images and (2) hierarchical classification of vertebral conditions (Fig. 2). This structured approach was designed to identify pathologies with clinical relevance and diagnostic accuracy.

#### Vertebrae extraction using pretrained segmentation network

The pretrained segmentation network utilized in this study is a multi-task U-Net, originally developed for detecting OVCFs and identifying vertebral levels [14]. This model was specifically employed for vertebrae segmentation from X-ray images. Designed to perform simultaneous learning tasks, it enables segmentation at the vertebral level while detecting the presence of OVCFs. Due to its reported performance in these dual tasks, this model was selected for our study. The pretrained weights provided by the original authors were utilized without additional fine-tuning on our dataset. This approach was chosen to leverage the model’s reliability and generalizability, particularly for anatomical structures like vertebrae, where significant domain-specific training had already been performed.

To optimize image quality and ensure network compatibility, a systematic pre-processing protocol was implemented for X-ray image segmentation. This process consisted of four sequential steps. First, raw X-ray images underwent intensity range clipping (1–99%) to remove outliers and normalize the intensity distribution, mitigating the influence of extreme pixel values that could compromise segmentation accuracy. Second, images were resized to a height of 1024 pixels using cubic interpolation to maintain structural detail fidelity. Third, zero-padding was applied to ensure standardized dimensions of 1024 × 1024 pixels, ensuring uniform input size. Finally, contrast enhancement was performed using Contrast Limited Adaptive Histogram Equalization (CLAHE) with a clipLimit parameter of 4, implemented via OpenCV [15]. This enhancement protocol improves the visualization of vertebral structures for subsequent segmentation.

**Fig. 3:**
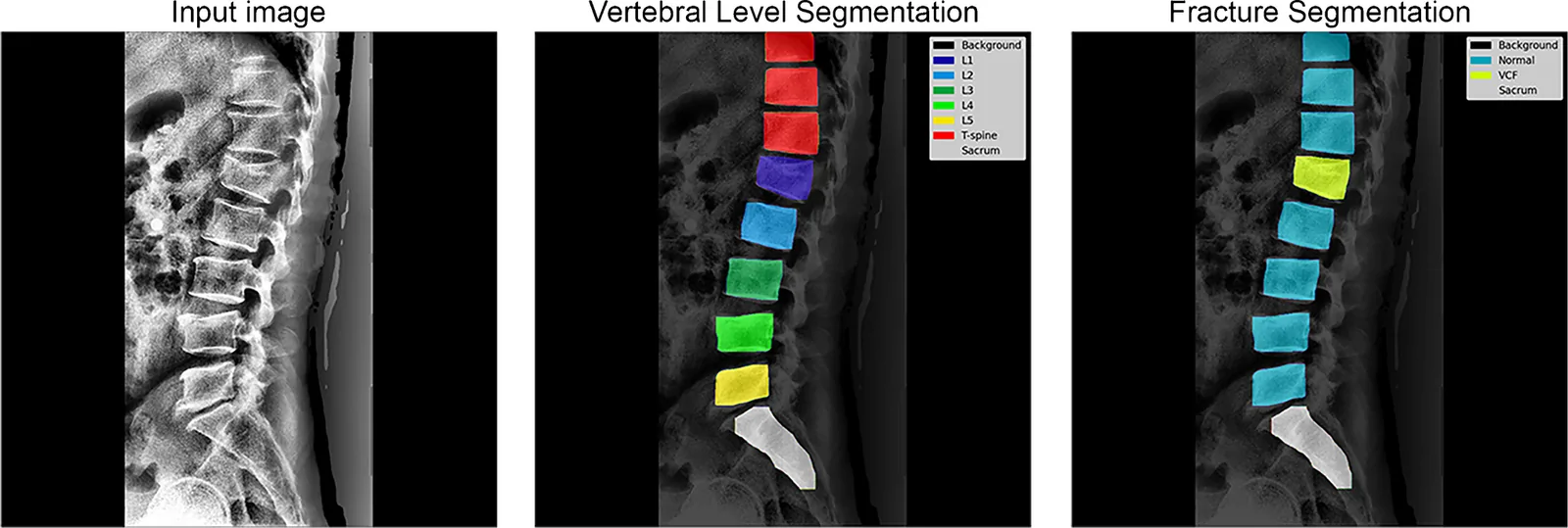
Workflow of automated vertebral segmentation. (**Left**) Raw input image. (**Middle**) Vertebral level segmentation identifying L1–L5. (**Right**) Fracture segmentation distinguishing compression fractures (yellow) from normal vertebrae (blue/cyan)

The lumbar spine segmentation process utilized the PSM described above, which was originally designed for both OVCF detection and vertebral level identification. This architecture allows for the concurrent optimization of these tasks by leveraging shared feature representations to improve segmentation performance. The model segments vertebrae patches from the X-ray images while identifying specific vertebral levels and potential OVCFs. The segmentation network extracts patches containing vertebrae (Fig. 3), ensuring consistent and reliable segmentation results for further analysis.

Our post-processing protocol consists of three sequential refinement steps to optimize vertebrae segmentation for classification analysis. First, we apply Skimage morphological operations to the segmentation masks, employing skimage.morphology.binary_erosion and binary_dilation functions with a kernel size of 5 [16]. This step reduces noise while preserving anatomical integrity through successive erosion and dilation operations. Next, individual vertebrae clusters are extracted from the refined segmentation masks. Finally, element-wise multiplication is performed between the original vertebrae images and their corresponding binary segmentation masks, effectively isolating vertebral regions by setting all external areas to zero. This structured refinement process ensures that the subsequent classification model receives optimally isolated vertebral features for analysis.

#### Hierarchical OVCF classification model

The classification methodology in this study employs the MobileNet V4 architecture, specifically adapted for differentiating vertebral conditions associated with OVCFs. Recent studies have demonstrated that MobileNet CNN approaches are highly effective and precise for bone fracture detection and classification [17]. Therefore, MobileNet V4 was selected to balance computational efficiency with diagnostic precision required for medical imaging applications. The architecture utilizes depthwise separable convolution layers, a design that reduces computational demands while preserving diagnostic accuracy, ensuring the feasibility of deploying a stable classification system in clinical settings without compromising analytical rigor.

The primary characteristic of the proposed methodology is its hierarchical classification framework, which reflects the progressive nature of vertebral pathology. Unlike conventional direct classification methods, this approach follows a two-stage process designed to reduce misclassification by reflecting the progression of vertebral healing. This hierarchical design aligns with previous findings indicating that specialized feature selection and grouping strategies can effectively distinguish subtle radiographic variations in vertebral fractures [18]. To evaluate the efficacy of this framework, we conducted a comprehensive comparative analysis using three distinct classification strategies:

1. End-to-End Classification (Single-stage): All vertebrae are simultaneously categorized into four groups (Normal, Chronic, Acute, or Indeterminate) in a single step. This serves as the baseline for comparison with hierarchical methods.
2. Normal-aligned Classification (Proposed Strategy): This approach initially groups cases based on the radiological similarity between chronic remodeling and normal bone to prioritize the early isolation of acute pathologies. In Stage 1, the model performs a three-class classification identifying Normal-Chronic (combined) vs. Acute vs. Indeterminate. Subsequently, in Stage 2, cases identified as Normal-Chronic are subdivided into distinct Normal and Chronic categories using a second-stage model.
3. Acute-aligned Classification (Comparison Strategy): This follows traditional fracture screening logic by first separating all fractures from normal vertebrae. In Stage 1, the model distinguishes between Normal, Fracture (combined Acute and Chronic), and Indeterminate cases. In Stage 2, the combined Fracture group is subsequently refined into separate Acute and Chronic categories.

The architecture leverages transfer learning principles through the integration of pre-trained MobileNet V4 weights. This approach allows for the application of established pattern recognition mechanisms while ensuring specificity for vertebral radiographic features. Standardization was maintained throughout both training and inference phases, with vertebral patches extracted via a segmentation network serving as consistent inputs for classification.

### Implementation details

This study utilized Python 3.10 for all data processing tasks, including pre-processing and post-processing. Deep learning model training and inference were conducted using PyTorch 2.1.0, with the latest version of MONAI (Medical Open Network for AI) to support medical imaging workflows. The deep learning training environment was equipped with two NVIDIA Quadro A6000 graphics processing units and 48 GB of DDR RAM, providing the necessary computational power to handle the complex model training requirements efficiently.

The use of ChatGPT (OpenAI) for English language editing of this manuscript is acknowledged. ChatGPT was not used for data analysis, research design, or scientific conclusions. All content was reviewed and edited by the authors, who take full responsibility for the final manuscript.

### Evaluation metrics

For comprehensive performance assessment, multiple evaluation metrics were employed, tailored to both vertebrae extraction and classification tasks. Vertebrae extraction performance was primarily evaluated based on accuracy in detecting lumbar spine vertebrae. For classification models, four standard metrics were utilized: sensitivity, specificity, accuracy, and F1-score. Given the inherent class imbalance in vertebral fracture datasets, where normal cases typically outnumber fracture cases, the F1-score was designated as the primary performance metric. The F1-score, being the harmonic mean of precision and recall, provides a more balanced assessment of model performance in imbalanced datasets, making it particularly suitable for evaluating our hierarchical classification approach in differentiating normal, chronic, acute, and indeterminate vertebral conditions.

### Statistical analysis

Model performance was evaluated on the held-out test set (20% of the total dataset). Sensitivity, specificity, accuracy, and F1-score were calculated for each classification strategy. Ninety-five percent confidence intervals for sensitivity, specificity, accuracy, F1-score, positive predictive value (PPV), and negative predictive value (NPV) were estimated by a clustered bootstrap (2000 resamples) in which radiographs were resampled with replacement, preserving the correlation among the five vertebrae obtained from the same radiograph.

### Visual explanation and feature representation

To interpret the model’s decision-making process, Gradient-weighted Class Activation Mapping (Grad-CAM) was employed to visualize the specific anatomical regions that most strongly influenced the classification outcomes. Additionally, t-Distributed Stochastic Neighbor Embedding (t-SNE) was utilized to project the high-dimensional latent feature representations into a two-dimensional space. This allowed for qualitative evaluation of cluster separation among normal, chronic, and acute vertebrae, providing visual validation of the proposed hierarchical classification strategies.

## Results

The hierarchical classification framework was evaluated through a quantitative analysis of both segmentation and classification performance. Segmentation accuracy was assessed using accuracy metrics against expert annotations on 260 lateral radiographs from the test dataset, while classification performance was measured through a comparative analysis of three distinct approaches using standard evaluation metrics, including sensitivity, specificity, accuracy, and F1-score.

### Vertebrae extraction result

The segmentation performance analysis demonstrated high accuracy in vertebral identification and delineation. The PSM achieved 98.9% segmentation accuracy, successfully identifying and isolating individual vertebral bodies from lateral X-ray images. This performance level was maintained even though the model was trained on an independent dataset, exhibiting high precision when applied to our clinical images. The consistent accuracy in vertebral segmentation established a reliable foundation for subsequent classification tasks.

The successful application of the pretrained model to our dataset validates its clinical utility. The consistent accuracy (98.9%) across different patient populations and imaging conditions indicates the stability and clinical applicability of the model. This reliable vertebrae extraction process proved crucial for generating standardized input patches for our hierarchical classification system, contributing to the overall performance of our OVCF detection framework.

### Classification result

The performance metrics of the three classification strategies—the conventional end-to-end approach and the hierarchical Normal-aligned and Acute-aligned strategies—are summarized (Tables 3 and 4).

**Table 3:** Performance of the end-to-end classification approach for vertebral fracture categorization

Strategy	Class	N	Sens (%)	Spec (%)	Acc (%)	F1 (%)	PPV (%)	NPV (%)
End-to-end classification	Normal	1056	84.9 (82.6–87.0)	89.1 (87.3–90.8)	85.8 (84.0–87.5)	90.4 (89.1–91.6)	96.7 (95.5–97.8)	58.9 (54.7–63.0)
	Chronic	73	64.0 (52.8–74.2)	90.2 (88.3–91.9)	88.7 (86.8–90.4)	38.0 (28.7–47.8)	97.2 (96.0–98.2)	97.8 (96.8–98.5)
	Acute	132	44.4 (36.1–52.9)	93.0 (91.4–94.5)	88.8 (87.1–90.4)	40.6 (32.8–48.5)	94.3 (92.9–95.6)	93.9 (92.4–95.1)
	Indeterminate	83	96.2 (90.1–99.0)	99.2 (98.6–99.7)	99.0 (98.3–99.6)	93.6 (88.4–97.0)	99.8 (99.3–100.0)	99.8 (99.3–99.9)

*Sens s*ensitivity, *Spec* specificity, *Acc* Accuracy, *PPV* positive predictive value, *NPV* negative predictive value. Values are point estimates with 95% confidence intervals, estimated by a clustered bootstrap (radiographs resampled)

**Table 4:** Performance of the hierarchical classification strategies (Normal-aligned and Acute-aligned)

	First stage								Second stage							
Strategy	Class	N	Sens (%)	Spec (%)	Acc (%)	F1 (%)	PPV (%)	NPV (%)	Class	N	Sens (%)	Spec (%)	Acc (%)	F1 (%)	PPV (%)	NPV (%)
Normal-aligned classification	Normal-Chronic	1129	91.9 (90.2–93.4)	86.1 (84.1–88.0)	91.0 (89.5–92.4)	87.8 (86.0–89.5)	97.2 (96.0–98.0)	67.0 (61.3–72.3)	Normal	1056	89.2 (87.3–91.0)	93.1 (91.6–94.5)	90.0 (88.4–91.6)	93.3 (91.9–94.6)	97.9 (96.8–98.7)	70.2 (65.4–74.5)
	Acute	132	91.0 (84.8–95.0)	82.1 (79.8–84.5)	91.2 (89.5–92.8)	81.2 (74.2–87.0)	35.6 (30.7–40.9)	98.8 (97.9–99.3)	Chronic	73	85.8 (75.4–93.0)	92.6 (90.8–94.3)	95.9 (94.4–97.3)	69.3 (58.2–78.9)	40.0 (32.8–47.9)	99.1 (98.5–99.5)
	Indeterminate	83	95.1 (89.0–98.3)	99.6 (98.9–100.0)	99.0 (98.2–99.6)	95.9 (90.8–98.6)	94.0 (86.8–97.4)	99.7 (99.2–99.9)	Acute	132	91.0 (84.8–95.0)	82.1 (79.8–84.5)	91.2 (89.5–92.8)	58.8 (52.0–65.3)	35.6 (30.7–40.9)	98.8 (97.9–99.3)
	-		-	-	-	-			Indeterminate	83	95.1 (89.0–98.3)	99.6 (98.9–100.0)	99.0 (98.2–99.6)	95.9 (90.8–98.6)	94.0 (86.8–97.4)	99.7 (99.2–99.9)
Acute-aligned classification	Normal	1056	94.9 (93.5–96.1)	95.5 (94.1–96.7)	95.0 (93.7–96.2)	96.9 (95.9–97.8)	98.9 (98.3–99.4)	76.4 (71.6–80.8)	Normal	1056	94.9 (93.5–96.1)	96.4 (95.1–97.5)	95.2 (93.9–96.3)	96.9 (95.9–97.8)	99.0 (98.1–99.4)	83.8 (79.7–87.5)
	Acute-Chronic	205	81.4 (75.3–86.5)	95.8 (94.6–96.9)	94.3 (92.9–95.6)	74.3 (67.8–80.3)	84.3 (78.7–89.0)	96.4 (95.2–97.4)	Chronic	73	45.9 (34.5–57.7)	94.0 (92.1–95.7)	91.4 (89.4–93.3)	36.6 (26.8–46.8)	30.5 (23.0–40.1)	96.8 (95.7–97.7)
	Indeterminate	83	91.8 (84.5–96.5)	97.7 (96.1–99.1)	97.2 (95.6–98.6)	84.1 (75.7–91.0)	77.6 (69.4–84.5)	99.3 (98.7–99.8)	Acute	132	46.1 (37.7–54.6)	95.9 (94.5–97.1)	91.6 (90.0–93.1)	48.7 (40.4–57.0)	55.0 (45.7–63.9)	94.2 (92.8–95.4)
	-		-	-	-	-			Indeterminate	83	97.0 (91.5–99.4)	99.0 (98.1–99.7)	97.3 (95.8–98.7)	84.1 (75.7–91.0)	86.5 (78.8–92.5)	99.8 (99.3–99.9)

*Sens* sensitivity, *Spec* specificity, *Acc* accuracy, *PPV* positive predictive value, *NPV *negative predictive value. Values are point estimates with 95% confidence intervals, estimated by a clustered bootstrap (radiographs resampled)

The end-to-end approach demonstrated variable performance across different vertebral conditions. For normal vertebrae, the model achieved a sensitivity of 84.9% (95% CI 82.6–87.0%) and a specificity of 89.1% (95% CI 87.3–90.8%), with an overall accuracy of 85.8% (95% CI 84.0–87.5%) (Table 3). However, performance dropped in the chronic and acute fracture groups, with sensitivities of 64.0% (95% CI 52.8–74.2%) and 44.4% (95% CI 36.1–52.9%), respectively, despite maintaining relatively high specificity (90.2% [95% CI 88.3–91.9%] and 93.0% [95% CI 91.4–94.5%]). Specifically, the model demonstrated high performance in the classification of indeterminate cases, achieving a sensitivity of 96.2% (95% CI 90.1–99.0%) and a specificity of 99.2% (95% CI 98.6–99.7%).

The Normal-aligned classification was implemented as a two-stage approach and showed improved overall performance. In the first stage, the method achieved strong results for both the Normal-Chronic classification (sensitivity 91.9% [95% CI 90.2–93.4%], specificity 86.1% [95% CI 84.1–88.0%]) and Acute classification (sensitivity 91.0% [95% CI 84.8–95.0%], specificity 82.1% [95% CI 79.8–84.5%], and a high negative predictive value [NPV] of 98.8% [95% CI 97.9–99.3%] at a test-set acute-fracture prevalence of 9.8% at the vertebra level). The second stage further refined these classifications, with Normal cases achieving 89.2% (95% CI 87.3–91.0%) sensitivity and 93.1% (95% CI 91.6–94.5%) specificity, and Chronic cases showing 85.8% (95% CI 75.4–93.0%) sensitivity and 92.6% (95% CI 90.8–94.3%) specificity (Table 4). The indeterminate category maintained excellent performance throughout both stages, achieving a sensitivity of 95.1% (95% CI 89.0–98.3%) and a specificity of 99.6% (95% CI 98.9–100.0%).

The Acute-aligned classification demonstrated the highest performance metrics for normal vertebrae identification, with 94.9% (95% CI 93.5–96.1%) sensitivity and 95.5% (95% CI 94.1–96.7%) specificity in the first stage. However, it showed lower performance for chronic and acute cases in the second stage, with sensitivities of 45.9% (95% CI 34.5–57.7%) and 46.1% (95% CI 37.7–54.6%), respectively, despite maintaining high specificity (94.0% [95% CI 92.1–95.7%] and 95.9% [95% CI 94.5–97.1%]). The indeterminate category consistently showed strong performance across both stages, achieving a sensitivity of 97.0% (95% CI 91.5–99.4%) and a specificity of 99.0% (95% CI 98.1–99.7%).

**Fig. 4:**
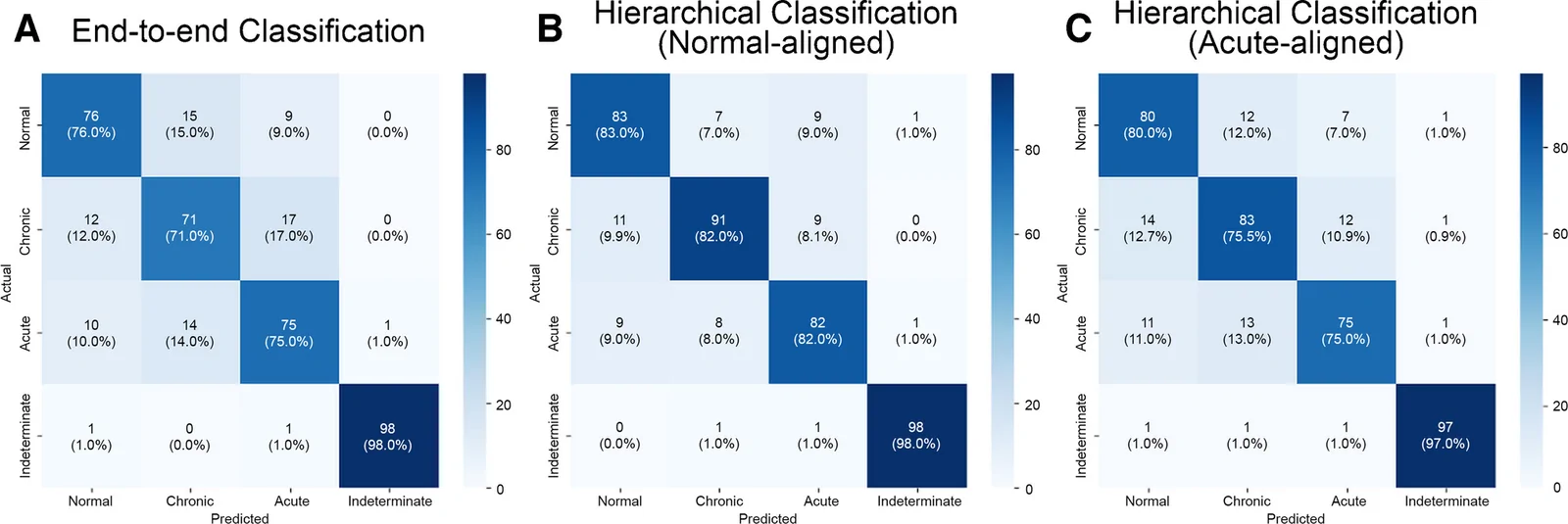
Confusion matrices of the three classification strategies. **A** End-to-end classification: A single-stage model trained to directly classify vertebrae into four categories: Normal, Chronic, Acute, and Indeterminate. **B** Hierarchical classification (Normal-aligned): A two-stage framework where Normal and Chronic cases are initially grouped (Stage 1) to prioritize the isolation of acute pathologies, followed by a second stage (Stage 2) to distinguish between Normal and Chronic vertebrae. **C** Hierarchical Classification (Acute-aligned): A two-stage framework that initially differentiates fractures (combined Acute and Chronic cases) from normal vertebrae (Stage 1), with a subsequent stage (Stage 2) designed to distinguish between acute and chronic fracture types

While each approach has its strengths, the Normal-aligned classification generally provided the most balanced performance across all categories, particularly in identifying chronic and acute cases while maintaining strong performance for normal and indeterminate vertebrae. The effectiveness of the Normal-aligned strategy is demonstrated quantitatively (Fig. 4), supporting the assumption that chronic OVCFs share more radiological characteristics with normal vertebrae than with acute fractures. Detailed performance metrics including accuracy and F1-scores for all categories are summarized (Tables 3 and 4).

To evaluate performance for the intended use as a patient-level triage tool, we additionally assessed each strategy at the patient level, classifying a patient as acute-positive when at least one vertebra was predicted as acute. In the held-out test set of 81 patients (46 [56.8%] with at least one acute fracture), the proposed Normal-aligned strategy detected all 46 acute patients, yielding a sensitivity of 100.0% (95% CI 92.3–100.0%) and an NPV of 100.0% (95% CI 83.9–100.0%), with no acute patient missed. This was achieved at the cost of 14 over-referrals among the 35 acute-negative patients, corresponding to a specificity of 60.0% (95% CI 42.9–75.0%). By comparison, the End-to-end and Acute-aligned strategies each missed 5 acute patients (sensitivity 89.1%); the Acute-aligned strategy reached the highest patient-level accuracy (86.4%) and specificity (82.9%) but did not achieve complete detection of acute patients. Full patient-level metrics for all three strategies are summarized (Table 5).

**Table 5:** Patient-level diagnostic performance of the three classification strategies

Strategy	Sens (%)	Spec (%)	Acc (%)	F1 (%)	PPV (%)	NPV (%)	Missed acute (FN)	Over-referred (FP)
End-to-end classification	89.1 (79.1–97.8)	62.9 (47.4–78.6)	77.8 (69.4–85.2)	82.0 (74.8–88.0)	75.9 (67.3–83.5)	81.5 (71.8–89.1)	5	13
Normal-aligned classification	100.0 (92.3–100.0)	60.0 (42.9–75.0)	82.7 (74.8–89.1)	86.8 (80.2–92.1)	76.7 (68.5–84.0)	100.0 (83.9–100.0)	0	14
Acute-aligned classification	89.1 (79.6–97.7)	82.9 (69.0–94.4)	86.4 (79.4–92.2)	88.2 (82.0–93.1)	87.2 (79.6–93.2)	85.3 (76.2–92.3)	5	6

*Sens* sensitivity, *Spec* specificity, *Acc* accuracy, *PPV* positive predictive value, *NPV * negative predictive value, *FN* false negative, *FP* false positive. A patient was classified as acute-positive if ≥ 1 lumbar vertebra was predicted as acute. 95% CIs by patient-level bootstrap (2000 resamples)

**Fig. 5:**
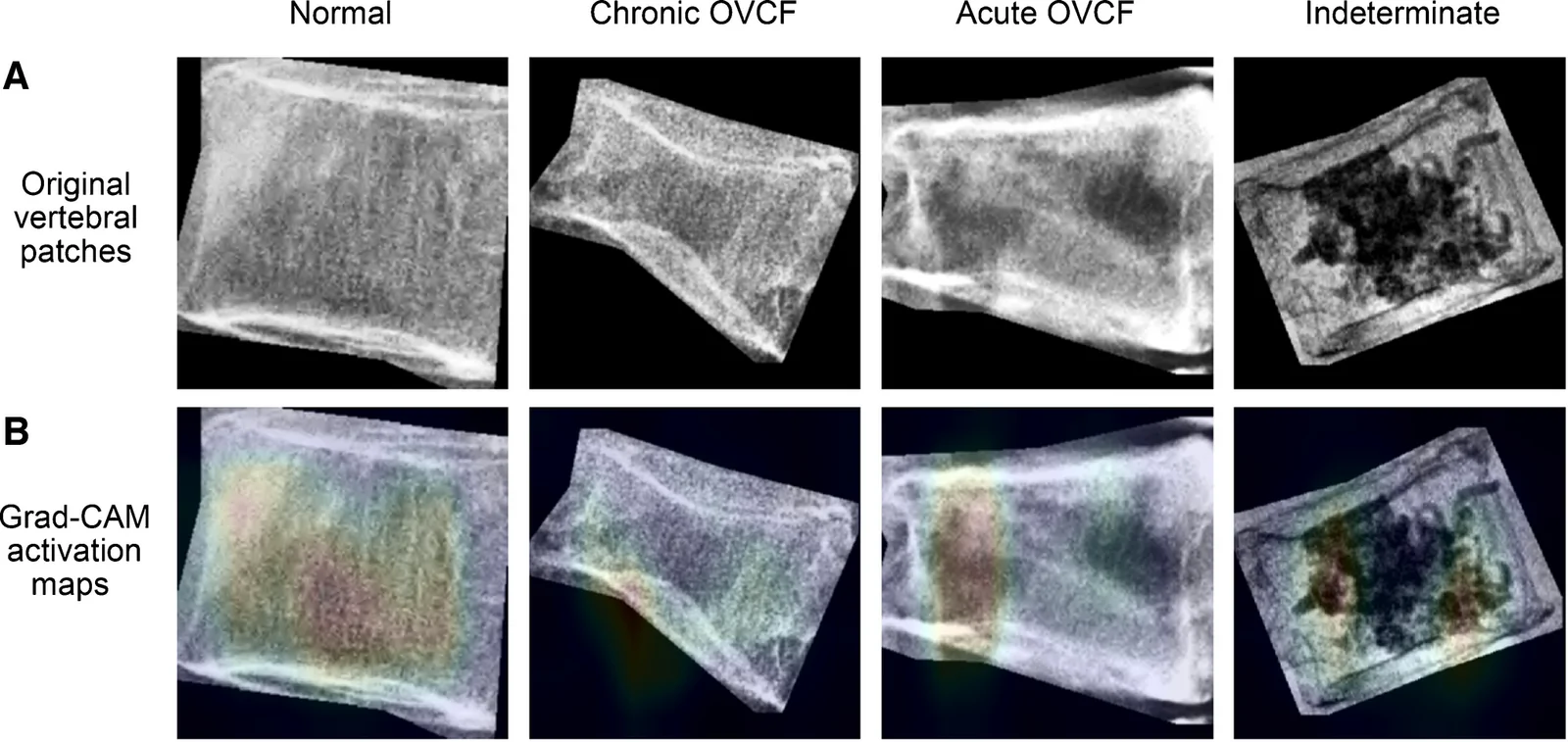
Representative vertebral examples across fracture categories. **A** Original vertebral patches from Normal, Chronic OVCF, Acute OVCF, and Indeterminate vertebrae. **B** Grad-CAM activation maps visualizing the anatomical regions most influential in the model’s classification decisions

Representative vertebral examples from each fracture category and corresponding Grad-CAM visualizations highlight the anatomical regions most influential in the model’s predictions (Fig. 5). These examples qualitatively illustrate how the model distinguishes fracture-related morphological features across normal, chronic, acute, and indeterminate vertebrae.

**Fig. 6:**
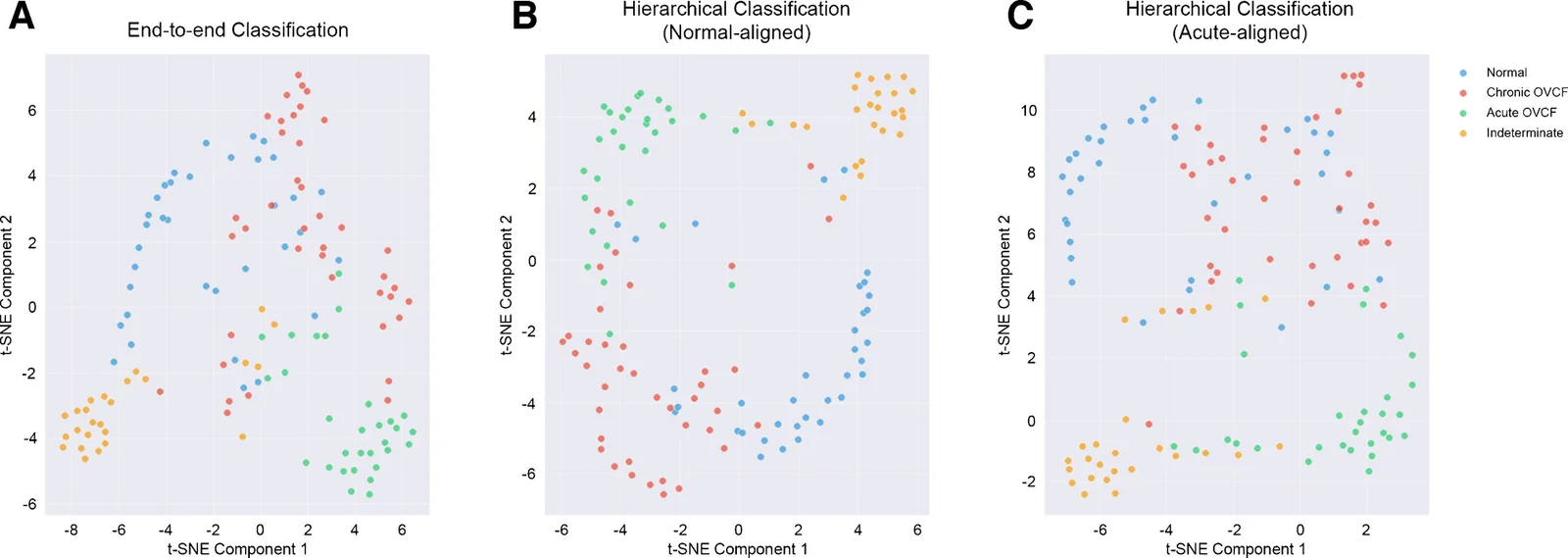
t-SNE visualization of latent feature representations from the three classification strategies. **A** End-to-end classification. **B** Normal-aligned hierarchical classification. **C** Acute-aligned hierarchical classification. The Normal-aligned approach (**B**) shows the most distinct cluster separation among Normal, Chronic, Acute, and Indeterminate categories. Chronic OVCFs cluster more closely with Normal cases than with Acute cases, validating their radiographic similarity and supporting the clinical rationale for the initial grouping strategy in the Normal-aligned framework

The t-SNE visualization of the feature space supports the efficacy of the Normal-aligned classification approach (Fig. 6). The dimensionality reduction analysis reveals distinct clustering patterns, with chronic OVCF cases demonstrating closer proximity to normal vertebrae compared to acute cases. This spatial distribution in the feature space corresponds with our quantitative findings, in which the Normal-aligned classification method achieved the highest performance metrics.

The observed feature distribution patterns demonstrated that chronic OVCFs retained more radiographic characteristics in common with normal vertebrae, while acute OVCFs formed a more distinctly separated cluster. This natural grouping tendency in the feature space aligned with the quantitative outcomes, in which the Normal-aligned classification achieved the highest performance.

## Discussion

This study aimed to develop a deep learning model capable of classifying lumbar OVCFs into distinct diagnostic categories: normal, chronic, and acute using only lateral X-ray images. Although advanced modalities such as MRI and CT are typically used to assess fracture acuity due to their ability to visualize marrow edema and cortical disruption, they are not always accessible in routine clinical settings. In many outpatient clinics and emergency departments, physicians must rely on plain radiographs for initial evaluation, despite the inherent difficulty of distinguishing acute from chronic fractures using X-rays alone.

Most existing AI-based studies on vertebral fractures have focused on CT or MRI, which offer high-resolution images suitable for assessing structural severity or marrow signal changes [19, 20]. While these studies have demonstrated strong performance, their practical application remains limited by issues such as cost, time, and accessibility. In contrast, lateral spine radiographs are more widely available and frequently used for first-line screening, emphasizing the need for tools that enhance their diagnostic value.

Recent work by Paik et al. demonstrated that deep learning can be applied to lateral X-rays for detecting vertebral compression fractures using a Mask R-CNN model trained with MRI-confirmed labels [8]. However, their model was designed to detect the presence of fractures rather than classify fracture type (i.e., acute vs. chronic). Building on these findings, our study addresses a more clinically relevant task by introducing a model achieving temporal classification distinguishing acute from chronic OVCFs using only X-ray data.

To achieve this, we developed a two-stage hierarchical classification model. Among the tested strategies, the Normal-aligned approach where normal and chronic cases were initially grouped yielded the most balanced performance across all categories. This model design was informed by real-world radiological patterns, where chronic fractures often resemble normal vertebrae due to partial healing or subtle collapse, whereas acute fractures tend to show more distinct deformities. By reflecting this clinical observation in model architecture, the Normal-aligned strategy improved discrimination between chronic and acute fractures while maintaining high overall performance.

Although our model included an indeterminate category to account for cases with postoperative changes or atypical anatomy, the primary clinical focus remained on the classification of fracture types relevant to clinical decision-making. Our results showed that the Normal-aligned strategy achieved high sensitivity (91.0%) and specificity (82.1%) for acute fractures, as well as strong performance for chronic and normal vertebrae. Importantly, the model also achieved consistently high F1-scores across categories, ranging from 58.8% to 95.9%. This suggests that the model maintained balanced performance even in the presence of class imbalance, particularly in detecting acute fractures that require timely clinical intervention.

This aligns with previous clinical observations indicating that morphologic changes such as vertebral height loss or kyphotic angle progression may correlate with fracture acuity [21], but to date, no algorithmic approach has demonstrated such classification capability directly from X-ray images.

Our model is among the first to apply a systematic AI-based framework for distinguishing acute and chronic OVCFs on lateral radiographs, achieving high sensitivity (91.0%), specificity (82.1%), and negative predictive value (NPV, 98.8%) for acute fracture detection. With an NPV of 98.8%, the model can serve as a highly effective triage tool or "gatekeeper" in emergency and outpatient settings. At the patient level, the Normal-aligned strategy identified all acute cases, achieving perfect sensitivity and NPV. Although this approach resulted in lower specificity and more unnecessary referrals than the Acute-aligned strategy, it is better suited for rule-out triage because missing an acute fracture has greater clinical consequences than additional referrals for advanced imaging. The Acute-aligned strategy may be preferred when balanced diagnostic performance is prioritized. It provides clinicians with the confidence to safely rule out acute fractures, thereby reducing unnecessary advanced imaging (e.g., CT or MRI) for patients with a low pre-test probability. By enabling more accurate initial assessment, the model has the potential to improve diagnostic workflows and support clinical decision-making in time-sensitive settings.

In previous X-ray–based deep learning studies for OVCF classification, vertebral labeling was performed solely based on radiographic assessment without CT or MRI reference [5, 9]. In the present study, all fracture classifications were confirmed against CT and/or MRI reports, which served as the definitive reference standard during the labeling process. This approach strengthens the reliability of the ground truth labels while preserving the practical advantage of an X-ray–only classification model.

However, several important limitations of this study should be noted. First, this was a retrospective study based on data collected from a single institution, which may limit the generalizability of the model across diverse clinical settings and populations. Because the cohort consisted of patients clinically suspected of OVCF, the acute-fracture prevalence in the test set (9.8% at the vertebra level) was higher than in a general population. As lumbar radiographs are usually obtained in symptomatic patients, this reflects the clinical setting in which the model would be used. PPV and NPV are prevalence-dependent and should be interpreted accordingly; in lower-prevalence settings the PPV would be lower and the NPV higher. Second, our study focused exclusively on the lumbar spine region for OVCF classification. While this focused approach allowed for detailed analysis of a crucial spinal region, it leaves open questions about the model’s applicability to thoracic vertebrae and other spinal segments where OVCFs commonly occur. Third, the current purely image-based approach does not incorporate potentially valuable clinical metadata such as patient age, bone mineral density measurements, or previous fracture history. Due to the strict anonymization protocol during the initial dataset construction, exact matching of individual clinical metadata with imaging identifiers was restricted, preventing correlation analysis between model performance and specific patient demographics. Additionally, detailed fracture morphologies, such as posterior wall involvement, were not systematically classified by the current algorithm, which remains a crucial area for future multimodal AI development. Fourth, the inherent resolution limitations of X-ray imaging may affect the model’s ability to detect subtle fracture characteristics, particularly in complex cases with overlapping conditions. Fifth, the lack of prospective validation in real-world clinical settings limits our ability to assess the system’s impact on patient outcomes and healthcare resource utilization.

Despite these limitations, our study provides early evidence that a hierarchical, X-ray–only AI model can assist in distinguishing fracture types relevant to treatment decisions. This framework may serve as a foundation for integrated diagnostic tools that operate effectively within time-constrained, resource-limited clinical environments, potentially improving workflow efficiency in primary assessment settings.

In conclusion, we propose a hierarchical classification approach for diagnosing lumbar OVCFs using only lateral X-ray images. Among the tested strategies, the Normal-aligned approach demonstrated the most consistent and balanced performance across clinically important fracture types—normal, chronic, and acute—achieving high diagnostic accuracy without requiring advanced imaging modalities such as CT or MRI. To our knowledge, this study presents one of the first systematic, fully image-based algorithms capable of classifying fracture acuity directly from plain radiographs. By enhancing the diagnostic utility of widely available X-ray imaging, this model may support early-stage clinical triage, reduce unnecessary advanced imaging, and facilitate more timely and appropriate management for patients with vertebral fractures.

## Data Availability

The datasets generated during and/or analyzed during the current study are not publicly available due to institutional patient privacy protocols but are available from the corresponding author on reasonable request. The pretrained segmentation model used for vertebral extraction is publicly available at https://github.com/SoyoungLee8/TLspine_FxLevel_Segmentation/tree/main [14]. The trained hierarchical classification model and associated code are not currently publicly available but may be shared upon reasonable request subject to institutional review.

## Abbreviations

AI: Artificial intelligence
CNN: Convolutional neural network
CT: Computed tomography
Grad-CAM: Gradient-weighted class activation mapping
MRI: Magnetic resonance imaging
NPV: Negative predictive value
OVCF: Osteoporotic vertebral compression fracture
PSM: Pretrained segmentation model
t-SNE: T-distributed stochastic neighbor embedding
